# Interplay of Biomechanical, Energetic, Coordinative, and Muscular Factors in a 200 m Front Crawl Swim

**DOI:** 10.1155/2013/897232

**Published:** 2013-03-17

**Authors:** Pedro Figueiredo, David R. Pendergast, João Paulo Vilas-Boas, Ricardo J. Fernandes

**Affiliations:** ^1^Centre of Research, Education, Innovation and Intervention in Sport, Faculty of Sport, University of Porto, Rua Dr. Plácido Costa 91, 4200-450 Porto, Portugal; ^2^Higher Education Institute of Maia (ISMAI), Avenida Carlos Oliveira Campos, 4475-690 Maia, Portugal; ^3^Center for Research and Education in Special Environments, Department of Physiology and Biophysics, University at Buffalo, 3435 Main Street, Buffalo, NY 14214, USA; ^4^Porto Biomechanics Laboratory, University of Porto, Rua Dr. Plácido Costa 91, 4200-450 Porto, Portugal

## Abstract

This study aimed to determine the relative contribution of selected biomechanical, energetic, coordinative, and muscular factors for the 200 m front crawl and each of its four laps. Ten swimmers performed a 200 m front crawl swim, as well as 50, 100, and 150 m at the 200 m pace. Biomechanical, energetic, coordinative, and muscular factors were assessed during the 200 m swim. Multiple linear regression analysis was used to identify the weight of the factors to the performance. For each lap, the contributions to the 200 m performance were 17.6, 21.1, 18.4, and 7.6% for stroke length, 16.1, 18.7, 32.1, and 3.2% for stroke rate, 11.2, 13.2, 6.8, and 5.7% for intracycle velocity variation in *x*, 9.7, 7.5, 1.3, and 5.4% for intracycle velocity variation in *y*, 17.8, 10.5, 2.0, and 6.4% for propelling efficiency, 4.5, 5.8, 10.9, and 23.7% for total energy expenditure, 10.1, 5.1, 8.3, and 23.7% for interarm coordination, 9.0, 6.2, 8.5, and 5.5% for muscular activity amplitude, and 3.9, 11.9, 11.8, and 18.7% for muscular frequency). The relative contribution of the factors was closely related to the task constraints, especially fatigue, as the major changes occurred from the first to the last lap.

## 1. Introduction

The goal of competitive swimming is to perform the race distance as fast as possible, for that swimmers must achieve their highest average velocity for that distance. Swimming velocity ($\overset{-}{\nu }$) is the product of the stroke rate (SR) and the distance moved through the water with each complete stroke cycle (SL) [1] and can be expressed as

(1) $$\begin{array}{c} \overset{-}{\nu }=\text{SR}\times \text{SL}. \end{array}$$

For the same $\overset{-}{\nu }\text{,}$ several combinations of SR and SL are possible and are a result of modifications of the time spent in different phases of the stroke cycle (interarm coordination), which can be measure in front crawl with the index of coordination (IdC; [2–4]). However, swimmers do not move at a constant velocity within each stroke cycle, and variations in the action of the arms, legs, and trunk result in intermittent application of force and lead to variations in the swimming velocity around the mean velocity within each stroke cycle. These intermittent movements and resultant variations in velocity increase the work done by the swimmer [5], compared to swimming at a constant velocity. The average velocity attained by the swimmer results from the average of the instantaneous velocity, resulting from intracycle velocity variation (IVV):

(2) $$\begin{array}{c} \overset{-}{\nu }=\nu_{\text{constant}}+\Delta \nu \left(t\right). \end{array}$$

In addition to these factors, maximal swimming velocity (${\overset{-}{\nu }}_{\max}$) depends on the maximal metabolic power of the swimmers (${\dot{E}}_{\text{tot-max}}$) and on their energy cost of locomotion (*C*):

(3) $$\begin{array}{c} {\overset{-}{\nu }}_{\max}=\frac{{\dot{E}}_{\text{tot-max}}}{C}, \end{array}$$

where ${\dot{E}}_{\text{tot-max}}$ can be computed based on measures/estimates of the aerobic, anaerobic lactic, and anaerobic alactic energy contributions and *C* (i.e., the amount of metabolic energy spent to cover one unit of distance, KJ·m^−1^). The *C* depends on biomechanical factors such as the mechanical efficiency (*η*_*m*_), the propelling efficiency (*η*_*p*_), and the mechanical work to overcome hydrodynamic resistance (*W*_*d*_):

(4) $$\begin{array}{c} C=\frac{W_{d}}{\left(\eta_{p}\times \eta_{m}\right)}. \end{array}$$

To assess *W*_*d*_ several methods have been proposed; however there is no agreement on the most valid method [6–8], and thus it remains difficult to determine active drag during a competitive event while preserving the ecology of the movement. On the other hand, propelling efficiency includes work done against drag and is defined as the ratio of useful mechanical work (*W*_*d*_) to total mechanical work (*W*_tot_):

(5) $$\begin{array}{c} \eta_{p}=\frac{W_{d}}{W_{\text{tot}}}, \end{array}$$

where in aquatic environments *W*_*d*_ is lower than *W*_tot_, since a fraction of the work produced by the contracting muscles is used to accelerate a variable amount of water backwards (wasted work) [9] and for the internal work [10]. The *η*_*p*_ includes *W*_tot_ and is dependent on the swimmers' technique and is velocity-dependent and affected by fatigue. In addition, mechanical efficiency is related to how muscles produce the mechanical work needed to sustain a given speed [10, 11]. Muscle efficiency arises from the range of either their force/length and/or force/speed relationships. Relations between force and iEMG have been used to estimate different efficiencies. Also, it has been suggested that the reduction in electrical efficiency with fatigue indicated that more motor units were recruited to generate the same amount of force compared with the nonfatigued muscle [12, 13]. However, the diagnostic value of the time domain analysis (iEMG) in muscle fatigue evaluation is considered to be more limited than that of the frequency domain analysis (Freq; [14]). So, to minimize the metabolic cost of high performance activities, the limbs must generate large power outputs while the muscles perform work at high efficiencies.

As described above, theoretical models have been developed that attempt to explain the influence of various factors on performance. In spite of the fact that velocity is common to the theoretical approaches, they cannot be combined due to incompatibility of terms and units. This has led to attempts at practical approaches, relating swimming performance to different anthropometrical, physiological, and biomechanical parameters [15–18]. This kind of research can be developed by comparing different competitive level swimmers, employing the neural network, computing cluster analysis, or developing statistical models from the swimmer's profile [19]. However, these studies have not theorized/assessed swimming performance completely using a biophysical approach, particularly at high swimming speeds [19–21]. The 200 m swim and freestyle swimming are the dominant competitive events and thus of great interest. Therefore, the purpose of this study was to determine the relative contribution of selected biomechanical (SL, SR, horizontal IVV, vertical IVV, *η*_*p*_), energetic (${\dot{E}}_{\text{tot}}$), coordinative (IdC), and muscular factors (iEMG and Freq) for the 200 m front crawl performance and each of its four laps. The approach used, in the absent of an appropriate theoretical approach, was a multivariate analysis of the important factors among those listed above that would account for the average swimming velocity in a 200 m front crawl swim and its component lengths, in well-trained swimmers. It was hypothesized that the biomechanical and energetic factors would be most important, with the coordinative and muscular factors also playing an important, but lesser, role.

## 2. Methods

### 2.1. Subjects

Ten well-trained swimmers (21.6 ± 2.4 yr) who were specialists in the 200 m front crawl event participated in this study. Height, arm span, body mass, and percentage of adipose tissue were 185.2 ± 6.8 cm, 188.7 ± 8.4 cm, 76.4 ± 6.1 kg, and 10.1 ± 1.8%, respectively. The subjects had an average of 11.9 ± 3.5 yrs of competitive experience. Their performances in the 200 m front crawl were 109.3 ± 2.1 s, which correspond to a mean velocity that represents 91.6 ± 2.1% of the mean velocity of the short course pool world record for men. The protocol was approved by the local ethics committee and followed the rules of the Declaration of Helsinki (2000). Swimmers were informed of the procedure, the potential risks involved, and the benefits of the study and then gave a written consent to participate. During the testing period, subjects were asked to adapt the intensity and the total volume of training to avoid stressful training programs. Swimmers' practiced with and were accustomed to all procedures, particularly swimming with the snorkel used for measurement of ${\dot{\text{V}}\text{O}}_{2}$.

### 2.2. Experimental Procedures

All tests were conducted in a 25 m indoor pool and each subject swam alone in the middle lane, avoiding pacing or drafting effects. Following a warm-up that consisted of a self-selected swim of about 1000 m, including some swimming with the snorkel, swimmers performed a 200 m maximum effort front crawl swim after a push start and using open turns without a glide. They were instructed to replicate their pacing and strategy used in competition. After 90 min of active rest, swimmers performed a 50 m front crawl test and twenty-four hours later a 150 m and a 100 m tests, with 90 min active rest interval between them. Together 50, 100, and 150 m tests were at the same swimming speed as in the previous 200 m paced by a visual light pacing system placed in the bottom of the pool. The pacing lights led the swimmers as the lights progressed down the pool with a flash every 5 m (TAR 1.1, GBK-Electronics, Aveiro, Portugal).

### 2.3. Data Collection and Analysis

#### 2.3.1. Biomechanical Factors

Each swimmer's performance was recorded with a total of six stationary and synchronized video cameras (Sony, DCR-HC42E, Tokyo, Japan), four below and two above the water. The calibration set-up, accuracy, and reliability procedures have been previously described in detail [22]. The twenty-one landmarks videoed (Zatsiorsky's model adapted by [23]) that define the three-dimensional position and orientation of the head, torso, upper arms, forearms, hands, thighs, shanks, and feet were manually digitized at 50 Hz using a commercial software package (Ariel Performance Analysis System, Ariel Dynamics, Inc., USA). The Direct Linear Transformation Algorithm [24] was used for three-dimensional reconstruction and a digital low-pass filter at 6 Hz was used to smooth the data. 

#### 2.3.2. Stroking Parameters

One complete stroke cycle (defined as the period between the instant of entry of one hand to the next instant of entry of the same hand) for each of the 50 m laps of the 200 m front crawl was analyzed. From these data, the center of mass position as a function of time was computed. The mean velocity ($\overset{-}{\nu }$) was calculated by dividing the horizontal displacement of the center of mass in one stroke cycle over its total duration. Additionally, the horizontal distance travelled by the center of mass during the stroke cycle was used to determine the stroke length (SL). The stroke rate (SR) was determined as the inverse of the time (seconds) to complete one stroke cycle, which was then multiplied by 60 to yield units of strokes per minute.

#### 2.3.3. Intracycle Velocity Variation

To determine and analyze the whole body centre of mass' IVV in the *x*, *y*, and *z* axes of motion, the coefficient of variation (CV = SD/mean) was computed as previously suggested [19, 22, 25].

#### 2.3.4. Propelling Efficiency

Propelling efficiency (*η*_*p*_) was calculated from the computed 3D hand velocity as the sum of the instantaneous 3D velocity of the right and left hand combined during the underwater phase of the stroke (3Du). The *η*_*p*_ was calculated from the ratio of the speed of the center of mass to the 3D mean hand velocity ($\eta_{p}=\overset{-}{\nu }/3\text{Du}$), since this ratio represents the theoretical efficiency in all fluid machines and has been used in swimming [18, 26].

### 2.4. Energetic Factors

#### 2.4.1. Total Energy Expenditure and Energy Cost of Swimming (*C*)

Oxygen uptake (${\dot{\text{V}}\text{O}}_{2}$) was recorded by means of the K4b² telemetric gas exchange system (COSMED, Rome, Italy), during the 200 m front crawl test. This equipment was connected to the swimmer by a low hydrodynamic resistance respiratory snorkel and valve system. This system was previously validated and widely used [15, 25, 26]. Expired gas concentrations were measured breath-by-breath and averaged every 5 s, to get the ${\dot{\text{V}}\text{O}}_{2}$ used in subsequent calculations. Net ${\dot{\text{V}}\text{O}}_{2}$ was calculated by subtracting the resting ${\dot{\text{V}}\text{O}}_{2}$ from the steady state ${\dot{\text{V}}\text{O}}_{2}$ measured during swimming. Before, and after, the 50, 100, 150, and 200 m tests, capillary blood samples (5 *µ*L) were collected from the ear lobe to assess rest and postexercise blood lactate (La_*b*_) using a portable lactate analyzer (Lactate Pro, ARKRAY, Inc.). Lactate was measured at 1, 3, 5, and 7 min after test, and the peak value was used for further analysis.

Since the 200 m front crawl energy contribution is supplied from the three energy sources [26–28], ${\dot{E}}_{\text{tot}}$ was calculated for each 50 m lap (for review see [28]):

(6) $$\begin{array}{c} {\dot{E}}_{\text{tot}}={\dot{\text{V}}\text{O}}_{2}+\beta \dot{\text{L}}{\text{a}}_{b}+\text{PCr}\left(1-e^{-t/\tau }\right), \end{array}$$

where ${\dot{E}}_{\text{tot}}$ is the total energy expenditure, ${\dot{\text{V}}\text{O}}_{2}$ is the aerobic contribution (calculated from the time integral of the net ${\dot{\text{V}}\text{O}}_{2}$ versus time), $\beta \dot{\text{L}}{\text{a}}_{b}$ is the net accumulation of lactate after exercise, *β* is the energy equivalent for lactate accumulation in blood (2.7 mL O_2_·mM^−1^·kg^−1^), PCr is the alactic contribution, *t* is the time duration, and *τ* is the time constant of PCr splitting at work onset (23.4 s). The contribution of each energy pathway was calculated for each lap, and on the basis of these data, ${\dot{E}}_{\text{tot}}$ was computed and *C* was calculated as the ratio between $\dot{E}$ and $\overset{-}{\nu }$.

### 2.5. Coordinative Factors

#### 2.5.1. Index of Coordination

The calculation of the index of coordination (IdC) requires the identification of key points in the stroke cycle [2, 4], specifically, (A) entry and catch of the hand in the water, (B) pull in the water, (C) push in the water, and (D) recovery out of the water. Each phase, within the stroke cycle, was determined from the swimmer's horizontal (*x*) and vertical (*y*) displacement of the hand noting the time corresponding to start and end of these phases for two arm stroke cycles previously digitized.

The IdC was calculated as the time gap between the propulsion (pull and push phases) of the two arms and expressed as a percentage of the duration of the complete arm-stroke cycle (sum of the propulsive and nonpropulsive phases (catch and exit phases)) [3, 29, 30]. IdC was the mean of IdC left and IdC right. 

### 2.6. Muscular Factors

The EMG signals of eight muscles (flexor carpi radialis, biceps brachii, triceps brachii, pectoralis major, upper trapezius, rectus femoris, biceps femoris, and tibialis anterior), which have been shown to have high activity during front crawl swimming [31, 32], were recorded simultaneously from the right side of the body using bipolar (interelectrode distance of 2.0 cm) Ag–AgCl circular surface electrodes. The skin of the swimmer was shaved and cleaned with alcohol and the electrodes with preamplifiers placed in line with the muscle's fibre orientation on the surface in the midpoint of the contracted muscle belly according to international standards [33] and covered with an adhesive bandage (OPSITE FLEXIFIX) [34, 35]. A reference electrode was attached to the body's patella. All cables were fixed to the skin by adhesive tape to minimize artifacts during swimming. Additionally, swimmers wore a total body coverage swimsuit (Fastskin, Speedo) to cover the electrodes and recording wires. The total gain of the amplifier was set at 1100 times with a common mode rejection ratio of 110. The data were sampled at 1000 Hz with a 16-bit analog to digital conversion and recording system (BIOPAC System, Inc) and stored on a computer for later analysis. An electronic flashlight signal synchronized with an electronic trigger marked simultaneously the video and EMG recordings, respectively, to synchronize EMG and video recordings. The EMG data analysis was performed using the MATLAB 2008a software environment (MathWorks Inc., Natick, Massachusetts, USA).

#### 2.6.1. iEMG

Raw EMG signals were band-passed (8–500 Hz), rectified to obtain the full wave signals, and smoothed with a 4th order Butterworth filter (10 Hz) for the linear envelope. The integration of the rectified EMG was calculated, per unit of time, to eliminate the stoke cycle duration effect (iEMG/T) and normalized to the maximum iEMG observed (signal was partitioned in 40 ms windows to identify the maximal iEMG) [36]. All iEMG values from the measured muscles taken in the mid-pool section for each 50 m were averaged. In addition, the average iEMG values of all 8 muscles were added together (iEMG) and used to represent the total electrical activity of swimming.

#### 2.6.2. Frequency Analysis

For the frequency analysis (Freq), spectral indices were calculated [37] and averaged. Spectral indices were obtained for each stroke, defined by video analysis, in the mid-section of the pool for each 50 m lap and they were averaged for each muscle. The spectral indices for each muscle were then averaged to determine the Freq factor used to represent spectral muscle information. Spectral indices have been shown to most accurately detect changes in muscle power during dynamic contractions [38], and their increases indicate fatigue [37, 38].

#### 2.6.3. Statistical Analysis

Mean (SD) computations for descriptive analysis were obtained for all variables (normal Gaussian distribution of the data was verified by the Shapiro-Wilk's test). A one-way repeated measures ANOVA was used to compare each factor along the 200 m. When a significant *F*-value was achieved, Bonferroni post-hoc procedures were performed to locate the pairwise differences between the means. All the statistical analysis was performed using STATA 10.1 (StataCorp, USA), with the level of significance set at 0.05. The effect size (*f*) for each variable was calculated in accordance with Cohen [39] to measure the magnitude of difference. 

### 2.7. Modeling of Performance

As described in the Introduction, absence of a theoretical model to combine the factors that contribute to swimming performance, a multiple linear regression was used to identify the relative contributions of factors that are associated with swimming performance. These, among the previous defined, factors are biomechanical (SL, SR, IVV*x*, IVV*y*, *η*_*p*_), energetic (${\dot{E}}_{\text{tot}}$), coordinative (IdC), and muscular (iEMG and Freq). This analysis was carried out for the 200 m front crawl velocity and then repeated for the velocities of each of the component 50 m laps to examine and compare the relative contribution of the factors in each segment of the swim. A common general multiple linear regression analysis was used to identify the weight of the factors identified as contributing to 200 m swim velocity and attaining 100% of the variance of the performance. The equation used for all the models tested was

(7) ν−=constant+kSL+kSR+kIVVx+kIVVy +kηp+kE˙tot+kIdC+kiEMG+kFreq,

where $\overset{-}{\nu }$ is the mean swimming velocity for the 200 m or the mean velocity of each 50 m lap that equals the sum of the model' constant with the factors, stroke length, stroke rate, intracycle velocity variation (*x* and *y*), propulsive efficiency, total energy expenditure, index of coordination, muscular activation, and spectral indices weighted by their specific beta coefficients (*k*). Both *C* and IVV*z* were not used in the model to limit the number of factors and they were reflected in ${\dot{E}}_{\text{tot}}$ and *η*_*p*_ or IVV*x*, IVV*y*, respectively. To better express the relative importance of the factor, the weights of the regression were converted to standardized regression coefficients (beta weights). 

## 3. Results

**Figure 1:**
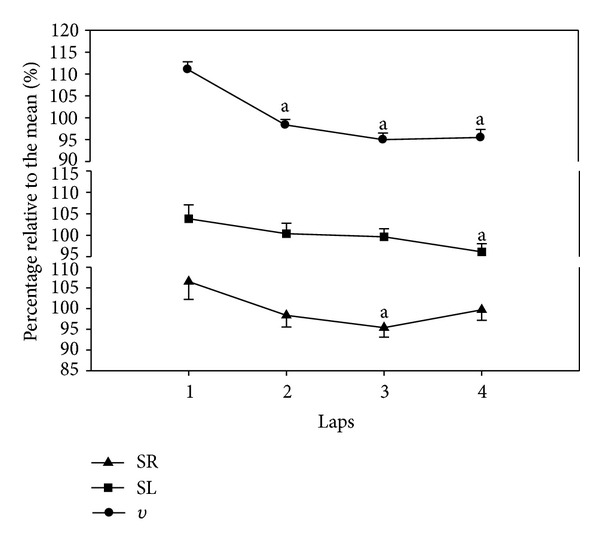
Mean (SE) values expressed as a percentage of the mean value for the 200 m front crawl for velocity (*v*), stroke length (SL), and stroke rate (SR) are plotted as a function of the 50 m laps. ^a^Significantly  different from the 1st lap.

Mean velocity for the total 200 m front crawl was 1.41 (±0.04) m·s^−1^.  Figure 1 shows the data for the average velocity of each 50 m lap, along with the observed stroke rate and stroke length, expressed as a percentage of their mean for the 200 m swim. The velocity in the first lap was faster than the average velocity but decreased below the average in the second lap, after which it remained constant (*F*_3,27_ = 24.72,  *P* < 0.001,  *f* = 1.27). Swimming velocity is the product of SR and SL, and they both decreased concomitantly with velocity (Figure 1). SR had a mean value for the 200 m of 38.41 (±3.05) cycles·min^−1^ but decreased across the swim, reaching a statistical difference after the third lap (*F*_3,27_ = 5.08,  *P* = 0.006,  *f* = 0.38). SL decreased below the mean for the 200 m of 2.20 (±0.14) m in lap 3 but reached significance only in the last lap (*F*_3,27_ = 4.55,  *P* = 0.01,  *f* = 0.33).

**Figure 2:**
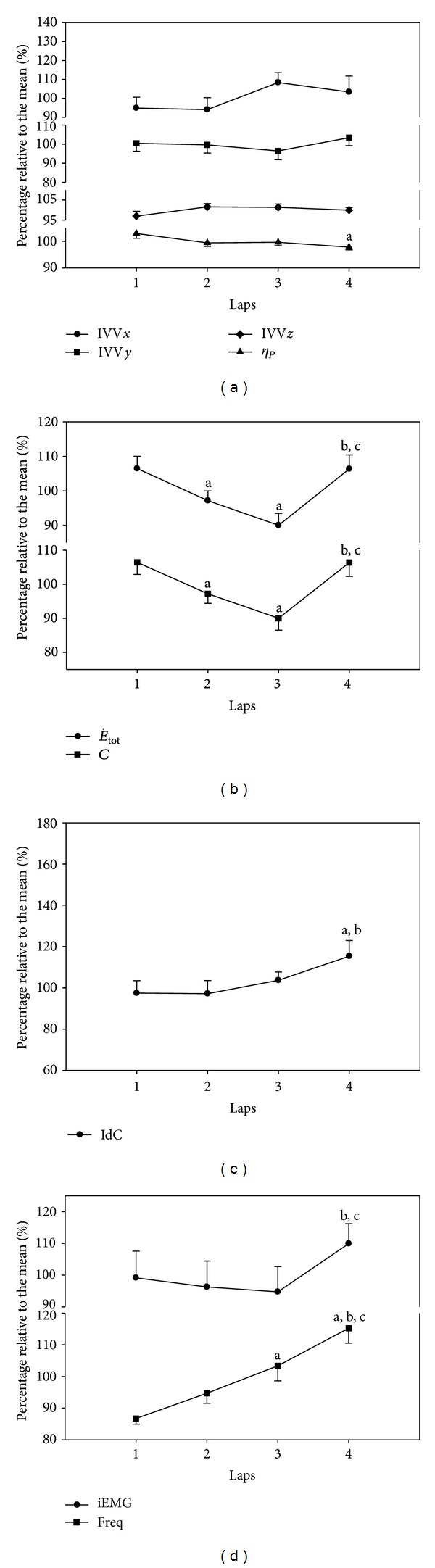
Mean (±SE) values for the percentage of the 200 m front crawl mean value for the (i) biomechanical factors: IVV for *x*, *y*, and *z* axes and  *η*_*p*_ (a); (ii) energetic factors  ${\dot{E}}_{\text{tot}}$  and  *C* (b); (ii) coordinative factor: IdC (c) and, (d) muscular factors: iEMG and Freq (d) for the 200 m front crawl event. ^a, b, c^Significantly different from the 1st, 2nd, and 3rd laps, respectively.

Figure 2 shows the four groups of factors identified as contributing to the 200 m front crawl swim (i.e., biomechanical, energetic, coordinative, and muscular). Biomechanical factor IVV (*x*, *y*, and *z*) (Figure 2(a)) mean values for the 200 m were 0.22 (0.03), 0.76 (0.08), and 0.83 (0.03), respectively. A stable pattern over the 50 m laps was observed (IVV*x*: *F*_3,27_ = 1.60,  *P* = 0.21,  *f* = 0.18; IVV*y*: *F*_3,27_ = 0.82,  *P* = 0.49,  *f* = 0.00; IVV*z*: *F*_3,27_ = 2.18,  *P* = 0.13, *f* = 0.24). Another biomechanical factor, *η*_*p*_, presented a mean value over the four laps of 0.42 (0.02) (Figure 2(a)), however, showed a significant reduction in the 4th 50 m lap (*F*_3,27_ = 6.64,  *P* = 0.002,  *f* = 0.41). Energetic factors, ${\dot{E}}_{\text{tot}}$ (*F*_3,27_ = 19.58,  *P* < 0.001,  *f* = 0.63) and *C* (*F*_3,27_ = 19.77,  *P* < 0.001,  *f* = 0.63) (Figure 2(b)), showed significant changes for the 50 m laps, with a mean of 80.11 (7.97) mml O_2_·kg^−1^·min^−1^ and 1.60 (0.16) KJ·m^−1^, respectively. The coordinative factor, IdC, presented a mean value of −14.94 (2.15)% (Figure 2(c)) and showed a significant increase in the 4th 50 m lap (*F*_3,27_ = 4.09,  *P* = 0.02,  *f* = 0.34). The two muscular factors, Freq (*F*_3,27_ = 30.40,  *P* < 0.001,  *f* = 0.89) and iEMG (Figure 2(d)), showed a significant increase (*F*_3,27_ = 4.22,  *P* = 0.01,  *f* = 0.22), in the last 50 m lap and the mean values were 1.97e^−14^ (0.22e^−14^) and 1.76 (±0.37), respectively.

The beta coefficients for all factors are presented in Table 1, for their contribution in the four laps to the 200 m velocity (upper half) and to the average velocity in each 50 m lap (lower half). Standardized coefficients from the multiple linear regression model showed that the contributions of the first and last 50 m laps velocity to the mean 200 m velocity were higher (26.1 and 30.8%, resp.) than the contributions of the second and third laps (21.7 and 21.4%, resp.) of the 200 m front crawl. The model had an *F*_4,5_ = 339.159, *P* < 0.001, *R* ^2^ = 0.996, and adjusted *R* ^2^ = 0.993 for these factors. These data are consistent with the changes in velocity shown in Figure 1. 

**Figure 3:**
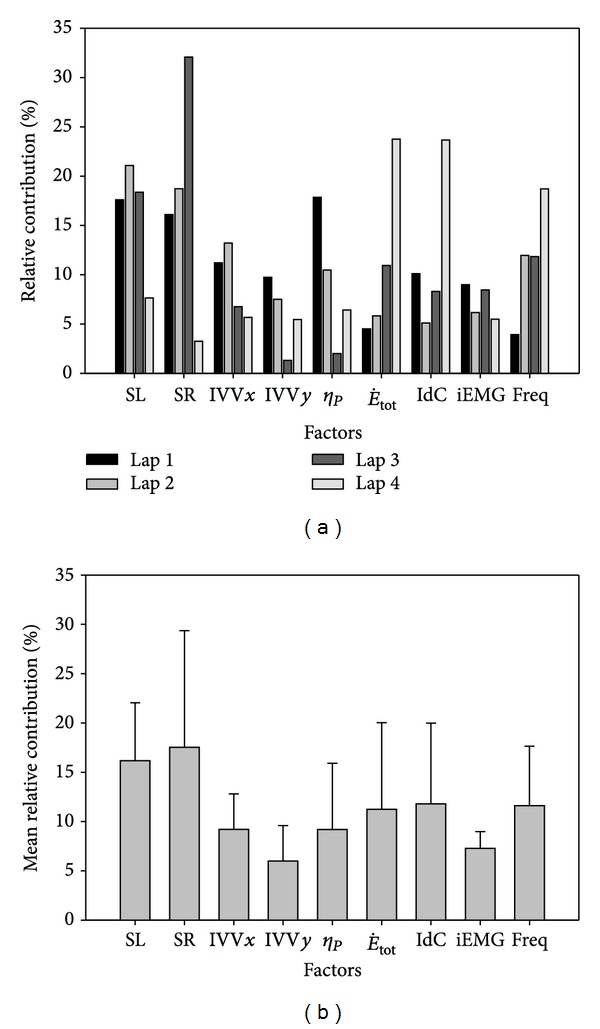
The percentage of the contributions of each factor in each lap for the 200 m swim performance (a) and mean percentages for all laps (b).

The biomechanical factors showed a great importance, manly the SL and SR (Figure 3) to the overall performance of the 200 m front crawl (16.2% and 17.5%, resp.). However, their contribution decreased in the final lap (from 17.6% and 16.1% to 7.6% and 3.2%, resp.). The SR had a very high contribution in the third 50 m lap (32.1%); concomitant with this, there was a great decrease in the contribution of the other biomechanical factors (6.7% for IVV*x*, 1.3% for IVV*y*, and 2.0% for *η*_*p*_), with the IVV*y* and *R* ^2^ factors increasing afterwards (5.4% and 6.4%, resp.). The ${\dot{E}}_{\text{tot}}$ contribution increases continually during the four laps (4.5%, 5.8%, 10.9% and 23.7%), while the IdC factor shows a “U” pattern with a large contribution at the beginning (10.1%), a decrease in the middle (5.1%), and then increase at the end of the swim (23.7% for the fourth lap). Relative to the muscular parameters (iEMG and Freq), iEMG appears to be quite stable (ranging from 5.5 to 9.0%), with only small oscillations, while the contribution of Freq increased over the length of the swim (from 3.9% in the first lap to 18.7% in the last lap).

**Figure 4:**
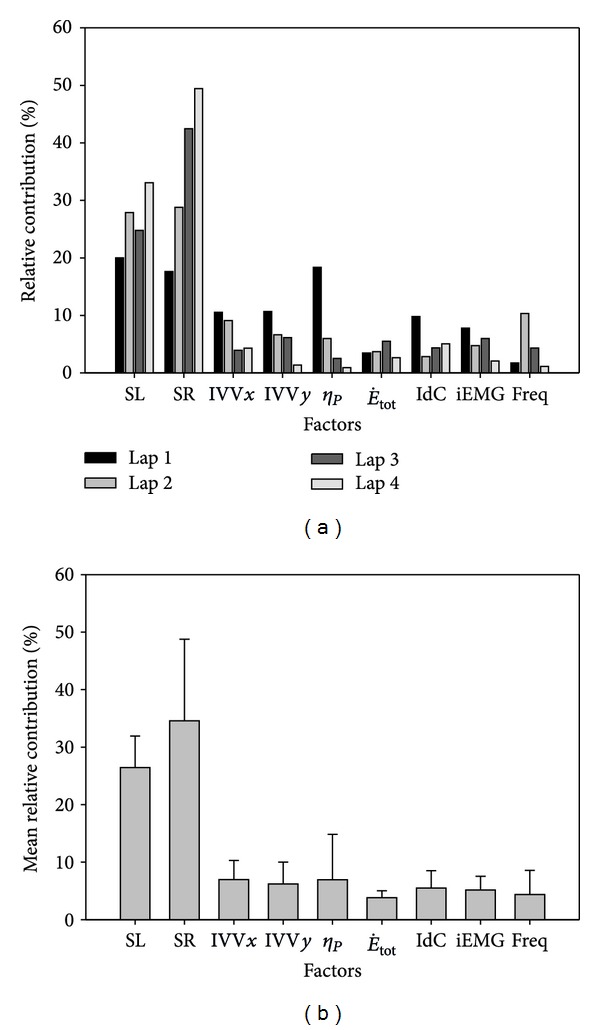
The relative contributions of the factors for the 50 m laps performances (a) and mean percentages for all laps (b) of the 200 m front crawl.

In Figure 4 the contributions of the relative importance of the factors used in the analysis for the average velocity in each lap individually are showed. The biomechanical factors (SL, SR, IVV*x*, IVV*y*, and *η*_*p*_) had a higher contribution (81.1%) than the energetic (${\dot{E}}_{\text{tot}}$, 3.9%), coordinative (IdC, 5.5%), and muscular (iEMG and Freq, 9.5%) factors. Within all the analyzed factors SL and SR showed the highest contribution (26.4% and 34.6%, respectively) the remaining ones (IVV*x*, IVV*y*, *η*_*p*_, ${\dot{E}}_{\text{tot}}$, IdC, iEMG and Freq) had a similar contributions (ranging from 3.8 to 6.9%). It should be noted that SL and SR are related mathematically with the *ν*. However, the contribution of each of these two factors for each 50 m lap performance showed that SL contribution decreased in the third lap (from 27.9% to 24.8%), in spite of its increase tendency over the four laps (from 20.0% in the first lap to 33.1% in the last lap), while the SR increased throughout the entire 200 m swim (from 17.6% to 49.4%). All the other factors used in the model showed a tendency to decrease their contribution from the beginning until the end of the swim, as the contributions of SL and SR increase.

## 4. Discussion

Although previous studies have evaluated the role of biomechanical [1, 40, 41], energetic [26, 27, 42], muscular [32, 35, 43], or coordinative [2, 4, 29] factors on the performance and others developed models to predict performance combining several factors [44], we are unaware of a study that examined their combined interactive effects as was performed in this study. The regression analysis performed was not intended to predict performance but to determine the contribution of the important factors to it. For the mean velocity of 1.41 m·s^−1^, the biomechanical, energetic, coordinative, and muscular factors were 58.1%, 11.2%, 18.9%, and 11.8%, respectively, with SL and SR factors explaining 33.7% of the 200 m mean velocity. A decrease in velocity during the second 50 m lap was observed, and then velocity was constant. Although the patterns were different, SL and SR decreased from the first 50 m and together accounted for the decrease in velocity. These changes in SL and SR are in agreement with previous studies [1, 29], showing the increase on the last lap of the SR to compensate for the SL decrease, in an attempt to maintain the velocity as high as possible. Also, the velocities that account for the major contribution to the overall performance of the 200 m front crawl were the first and last lap velocities, suggesting two important stages during this particular event. On the first lap, the highest velocities are achieved and on the last lap the consequences of fatigue were felt, and although velocity was constant, the contribution of the factors determining it changed. Among the 50 m laps, the contribution of biomechanical, energetic, coordinative, and muscular factors was on average 81.1%, 3.9%, 9.5%, and 5.5%, respectively, and 61% of the biomechanical contribution was attributed to the SL and SR.

### 4.1. Biomechanical Factors

Stability in the IVV (*x*, *y*, and *z*) was observed over the four laps, as previously reported by Psycharakis et al. [40] and Alberty et al. [29]. IVV stability seems to be related with a coordinative adaptation of the upper limbs, as IdC changes along the effort, as well as the SR, mainly in the last 50 m lap [45, 46]. IVV (*x*, *y*) accounted for 15.2% of the variability of the 200 m swim and 13.2% for the 50 m laps. In spite of the stability of IVV*x*, the *η*_*p*_ decreased in the last lap likely due to fatigue, as fatigue has been shown to evolve during the 200 m front crawl [26, 29]. Also, it indicates a reduction in stroke technique quality; at the end of effort [47], higher lactate accumulation occurs [48], as well as neuromuscular fatigue [49]. As a result, *η*_*p*_ accounted for 9.2% of the variability of the 200 m swim and on average 6.9% for the 50 m laps individual performance.

### 4.2. Energetic Factors

${\dot{E}}_{\text{tot}}$ and *C* decreased over the second and third 50 m laps, concomitant with the velocity decrease. However, taking into account the determinants of *C* (the hydrodynamic resistance and the propelling efficiency) and since *η*_*p*_ decreased due to the development of fatigue, *C*, and thus ${\dot{E}}_{\text{tot}}$, increased in the last lap, which is in agreement with previous studies [26, 50]. ${\dot{E}}_{\text{tot}}$ accounted for 11.2% of the variability of the 200 m swim and on average 3.9% for the 50 m laps. 

### 4.3. Muscular Factors

The assessed muscular factors revealed in spite of swimming at maximum effort that the observed muscles were involved at a submaximum level, as amplitude increased and frequency decreased (i.e., increase in the spectral indices), as previously reported for amplitude [43] and frequency [51] for a 4 × 50 m test simulating the 200 m front crawl, and also shown for the 100 m swim [32]. Similar results were observed in other sports activities [52, 53]. Most of these studies interpreted the increase in the EMG activity amplitude as increased motor units recruitment and increased motor units synchronization, as well as the decrease in muscle fiber conduction velocity, due to an accumulation of metabolic products. The iEMG and Freq factors contributed by 7.3% and 11.6%, respectively, to the variance of the 200 m swim and on average by 5.1% and 4.4%, respectively, to the 50 m laps.

### 4.4. Coordinative Factors

As velocity and the SL-SR ratio changed, interarm coordination adapted, with an increase in IdC in the final stages of the 200 m event. This observation is consistent with the development of fatigue as reported previously [29, 30]. Interlimb coordination is adapted, as an optimization mechanism to obtain as much speed as possible in face of constraints imposed [54], showing that an effective front crawl swimming technique must be sufficiently flexible and adaptable [55]. This factor (IdC) accounted for 18.9% of the variance of the 200 m swim performance and on average for 5.5% of the 50 m laps. 

### 4.5. Interplay among Factors

**Figure 5:**
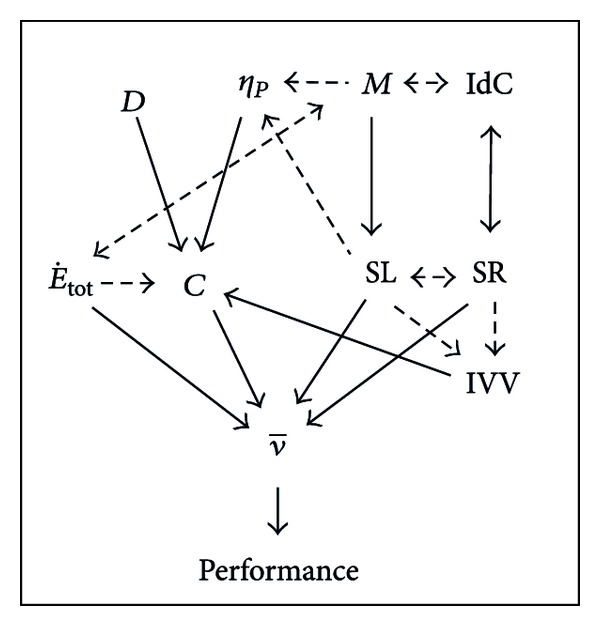
The relationship between biomechanical, energetic, coordinative, and muscular factors to performance in competitive swimming. *C*: energy cost, ${\dot{E}}_{\text{tot}}$: energy expenditure, IVV: intracycle velocity variation of the center of mass, IdC: index of coordination, SR: stroke rate, SL: stroke length, $\overset{-}{\nu }$: mean swimming velocity, *D*: hydrodynamic drag, *η*_*p*_: propelling efficiency, and *M*: muscular activation and frequency.

A theoretical framework for the interaction of the biomechanical, energetic, coordinative, and muscular factors is presented in Figure 5 and used in the subsequent discussion.

The biomechanical factors had the highest contribution to the 200 m front crawl and also to each 50 m lap mean velocities, where together they accounted for up to 33.7% and 61.0%, respectively. These contributions are understandable, as the product of two of these factors (SL and SR) determines swimming velocity [1]. The contributions of SL and SR to the total performance are very important to achieve high velocities however their contributions decreased during the swim, which suggested that several other factors had increased importance in determining the last 50 m lap velocity (see Figure 5). The contribution of SL showed a higher contribution than SR in the last 50 m. This observation is supported by a study of Craig et al. [1], where the best swimmers in the 200 m front crawl could maintain higher SL distances at the end of the event, in spite of having similar SRs. 

Changes in SL and SR are associated with IVV*x* and IVV*y* (see Figure 5); however, the latter showed a stable pattern over the 200 m. In spite of its stability, IVV*x* showed a decreased contribution over the length of the swim. IVV*y*'s contribution decreased even more than IVV*x* in the third lap, and then it increased in the fourth lap. Relative to the individual lap performances, *η*_*p*_ had similar mean contributions to velocity as IVV*x* and IVV*y*, and all of them decreased from the beginning to the end of the swim. IVV*x*'s and SL contributions to the 200 m performance showed a similar pattern, which could be explained by the increased time between propulsive phases as SL decreases and SR increases [2–4]. This change is also associated with a decrease in *η*_*p*_ (see Figure 5) [9, 18, 26] and increase in the IVV*x* and ${\dot{E}}_{\text{tot}}$ [56]. This can be explained as a smaller IVV will lead to a lower energy cost, for example, if two swimmers swim at equal mean velocity but the IVV = 0 in swimmer 1 and in swimmer 2 IVV > 0, then mean power of swimmer 1 will be *ν* ^3^ but in swimmer 2 it will be >*ν* ^3^, as ${\dot{E}}_{\text{tot}}$ has the same relation with *ν* [17, 27]. Supporting this concept, it was found that swimming with hand paddles, which increases *η*_*p*_ and SL [57], results in decrease of IVV*x* and increase of IdC [58]. On the other hand, IVV*y* and *η*_*p*_ showed a contribution pattern that was inverse of that of SR.

IVV*y* can be linked to the medial-lateral hand movements that account for vertical displacement changes suggesting great importance of the sideways movements during the stroke's propulsive actions, which have been highlighted by previous studies (for review see [59]) and are decreased with higher SRs. In addition, as *η*_*p*_ is SL-related [18, 26], its contribution to the variance in performance decreases when the SR contribution increases. The similar pattern observed for IVV*y* and *η*_*p*_ seems to confirm the possible link between IVV*y* and the sideways hand pattern motion, which resulted in a high *η*_*p*_. This may also account for the larger contribution of *η*_*p*_ than IVV*y*. Propelling efficiency's decreasing contribution to the laps performance might be linked to reduced muscles force production during the stroke due to fatigue. It is likely that a reduced muscle force production occurs, as indicated by the changes in EMG factors, and the swimmers became unable to sustain the initial SL [1, 60], as observed in this study. The spectral indices (Freq) have been suggested as one of the first indicators of fatigue [37, 38]. The SL and *η*_*p*_ decreases shown in this study are likely the result of fatigue developing toward the end of the 200 m swim. 

As the biomechanical factors show a decreased contribution to the variance of the 200 m in each 50 m swim performance between the first and the last laps, other factor's contributions must increase (see Figure 5). This was the case for the energetic and coordinative factors. Over the 50 m laps, the contribution of ${\dot{E}}_{\text{tot}}$ to the overall performance increased; thus the swimmer's capacity to deliver higher energy expenditure became more important over the 200 m. Swimmers can have the same time splits for the 50, 100, and 150 m, but if ${\dot{E}}_{\text{tot}}$ cannot be increased to match the increase in C in the last 50 m, velocity cannot be sustained. The contribution of ${\dot{E}}_{\text{tot}}$ in the three final laps is similar to that of IdC, which could be explained by the swimmer naturally adopting a movement pattern to minimize his metabolic energy expenditure [61, 62]. 

The reduction in *C*, and thus ${\dot{E}}_{\text{tot}}$, in laps 2 and 3 may involve the process of self-optimization [62] which occurs to overcome the constraints imposed, in this case by the fatigue task constraint [3]. The IdC factor had the inverse pattern of contribution to performance in the first three laps compared to that of SL. As indicated by previous studies, based on the dynamical theories of motor organization, stroke rate is the first determinant of motor organization in swimming [3] and it has an inverse relation with SL [4, 60]. As SR and IdC are associated with each other (see Figure 5), IdC had a higher contribution to first 50 m lap, as was the case in overall performance analysis. This is likely due to the direct relationship between IdC and velocity that has been suggested [2, 4]. After the first 50 m, the contribution of IdC starts to decrease, as a result of the decrease in velocity and SL, until the development of fatigue, which resulted in an increase in the contribution of the SR to a greater extent than SL. When strokes are closer to one another, or overlap, this has the effect of increasing the average propulsive force while the mean force per stroke is maintained [30]. These changes in stroke patterns increase the contribution of IdC in the latter stages of the 200 m swim, which also has previously been shown [29]. 

The increased ${\dot{E}}_{\text{tot}}$ contribution to overall performance reported in this study is related to the changes in the balance of the three energy pathways (aerobic, anaerobic lactic, and anaerobic alactic) as a function of time as previously reported [26–28]. The increase in contribution of the anaerobic lactate contribution and resultant lactate accumulation by the end of the effort [26] contribute to the explanation of the decrease in SL and *η*_*p*_. These changes are consistent with the deterioration of stroke mechanics observed by other authors [50, 60]. The reduced SL and increased Freq are associated with muscle fatigue most likely brought about by high lactate levels and reduced muscle glycogen [63]. This conclusion is supported by the suggestion that the increase in blood lactate concentration may change the stroking strategy significantly [60] and thus IVV*y* and *η*_*p*_. These deviations from the optimal combination of SL and SR result in a significant increase in energetic demand (see Figure 5), suggesting that minimizing energy cost may be an important factor contributing to cadence determination in cyclical forms of locomotion [62]. Supporting this, swimmers preferred to swim front crawl at the lowest SR (or the longest SL) that does not require an increase in oxygen uptake [64], as a significant decrease in the preferred SR, for example, determines the decline in time limit exercise duration [65], which might be caused by an unusual muscular recruitment.

The increase in ${\dot{E}}_{\text{tot}}$, particularly of anaerobic lactic contribution, in the final lap due to muscle fatigue is generally (although not exclusively) attributed to the reduced muscular fibre conduction velocity, which is causally related to a decrease in the pH [66]. Although pH was not directly measured, high values of blood lactate concentration collected after the 200 m swim [26, 27, 67] implied a significant pH decrease during swimming. As muscles fatigue, power output is reduced during the swim [41], as is the case for SL. Since the SL is an index of propelling efficiency [18, 59], *η*_*p*_ should decrease, as was observed in this study. The resultant deterioration of stroke mechanics in fatigued subjects is expected to lead to a progressive increase in the energy cost of swimming (see Figure 5), as was observed in this study. However, to maintain the total mechanical power output as Craig et al. [1] have shown for races of 200 m and longer, the distance per stroke tends to decrease as fatigue develops and SR has to increase to compensate to maintain the speed constant, or if SR and ${\dot{E}}_{\text{tot}}$ cannot be increased velocity decreases, which happens in this study in the second lap. In addition, increases in muscle activity can lead to decreases in efficiency (see Figure 5) with no increase in power output if the muscle coordination is inappropriate [11]. Muscle coordination changes due to fatigue in swimming have been shown [35].

In the first lap, the contribution of the SL is higher than the SR, but in the last lap SR is greater suggesting fatigue in the last lap, which is supported by the EMG data (see Figure 5). The muscular factor iEMG (amplitude analysis) has a tendency to decrease its contribution to the overall performance of the 200 m during subsequent 50 m laps. In the first 50 m lap, the highest contribution of the iEMG over the 200 m could be associated with the high velocity and also a higher contribution of the SL, which is linked to higher force production (see Figure 5) [68]. This would also be associated with a higher power output and velocity, as was the case for the first lap and concomitant with the high contribution observed. On the third lap, the iEMG contribution increases after its decrease in the second lap, which might explain the decrease in the absolute value of SR in this particular lap. This is supported by the higher SR in this lap that was associated with higher EMG activity [69] and its increase in contribution. Also, additional recruitment or increased synchronization of muscle fibers as a result of submaximal fatigue [70] most likely explains the reduced contribution in the last 50 m. If the velocity is an indicator of the power output and it was stable in the last three laps, mechanical efficiency and concomitant efficiency of the electrical activity was decreased, as the iEMG increased, if the electrical efficiency the ratio force to iEMG was considered [12, 13].

In spite of these associations described above, the relationship between iEMG and force is not linear and the diagnostic value of the time domain analysis (iEMG) in muscle fatigue evaluation is considered to be more limited than that of the frequency domain analysis (Freq) [14]. Freq showed a higher contribution to the 200 m swim than iEMG in the mean values and for the second, third, and fourth laps. These higher contributions might be explained by the ${\dot{E}}_{\text{tot}}$ absolute values and contributions, as ${\dot{E}}_{\text{tot}}$ absolute value is higher on the first lap, because of the higher velocity when swimmers are not fatigued. However, after the first lap velocity starts to decline, as did ${\dot{E}}_{\text{tot}}$, and a statistical stability during the second and third laps is being maintained. Freq's contribution increases during these two laps. As swimmers reach the fourth lap, Freq increases and SL decreases suggesting the presence of fatigue; ${\dot{E}}_{\text{tot}}$ increases in both absolute value and contribution to velocity in spite of the constant velocity. The contribution of the increased Freq over the swim distance attained a similar contribution to the overall performance as did the energetic and coordinative factors.

 For the mean velocity in each lap, both iEMG and Freq present a similar mean contribution; however their pattern of change over the laps is different. The iEMG has its highest contribution on the first lap, whereas Freq has a small contribution. However, Freq is higher, and iEMG lower, in the last lap. This can suggest that at the beginning of the effort higher muscular activation is needed to recruit more fast-twitch muscle fibers and achieve the higher SR at this stage. In the second lapes, the contribution of Freq surpasses that of iEMG, and after this, it decreases constantly until the end of the 200 m effort. The decreased contribution of iEMG is contrary to the increase in absolute values relative to the mean value for 200 m. This pattern of changes is similar to the decrease in spectral parameters that indicate the evolvement of fatigue. As higher *η*_*p*_ requires higher effective application of propulsive force, the decrease of the contribution of iEMG might be associated with a decrease in the contribution of *η*_*p*_ and be associated with muscle fatigue. 

Notwithstanding the results and discussion, as well as the combined interactive effects of performance influencing factors on several research fields in well-trained swimmers, the approach used has some limitations that have to be acknowledged. The regression analysis was not intended to predict performance, only to determine the contribution of the factors, and the variables used represent discrete and extremely important outcomes, each of them for the understanding of the swimming performance and aquatic human locomotion. The relation between the number of variables and subjects evaluated was poor, which may influence the results of the analysis performed, over- or underestimating the contribution of the factors. 

## 5. Conclusion

The swimmers in this study had the highest velocity in the first lap of the 200 m swim. The factors contributing to this were a balance of SL, SR, *η*_*p*_, IVV*x*, IdC, and iEMG, denoting particular importance for the biomechanical factors (SL, SR, and *η*_*p*_), as this first lap is done comfortable enough, without fatigue constraints. From the second through the fourth lap, although the velocity was similar, dynamical changes occurred in the importance of the contributing factors, especially in the fourth lap. In this last, the contributions of Freq and IdC were high and suggest fatigue of the muscles used in swimming, resulting in a high contribution of ${\dot{E}}_{\text{tot}}$ and lower contribution of *η*_*p*_. These data may suggest swimming at a uniform velocity, to avoid the effects of fatigue, and/or training to increase ${\dot{E}}_{\text{tot}}$ and muscular endurance. 

## Figures and Tables

**Table 1:** The beta coefficients (*k*) determined to identify the importance of the factors included in the multiple linear regression models computed for the mean for the overall 200 m performance, as well as for each individual performance lap.

		SL	SR	IVV*x *	IVV*y *	*η*_*p*_	${\dot{E}}_{\text{tot}}$	IdC	iEMG	Freq	Constant
200 m performance	Lap 1	−1.10	−0.04	4.04	−1.40	11.55	0.01	0.05	−0.32	0.01	0.89
	Lap 2	5.90	0.26	−14.15	−3.52	−28.08	0.04	−0.08	−0.64	−0.04	−2.12
	Lap 3	0.20	0.02	0.25	−0.02	−0.16	0.002	−0.006	0.03	0.001	0.05
	Lap 4	−0.13	−0.002	0.21	0.12	−0.97	0.005	−0.02	−0.04	0.002	1.04

Each 50 m performance	Lap 1	−6.52	−0.25	19.87	−8.02	62.06	0.03	0.26	−1.44	0.02	4.29
	Lap 2	1.32	0.07	−1.65	−0.52	−2.72	0.004	−0.01	−0.08	−0.01	−1.75
	Lap 3	0.63	0.05	0.35	−0.18	−0.49	−0.002	−0.01	0.05	−0.001	−1.59
	Lap 4	0.50	0.03	−0.15	−0.03	−0.12	0.001	−0.004	−0.01	−0.0001	−0.92
